# Phosphate Transporters Mediate the Uptake of Monothioarsenate

**DOI:** 10.1111/pce.70400

**Published:** 2026-01-28

**Authors:** Sebastian Haider, Sylvia Hafner, Britta Planer‐Friedrich, Stephan Clemens

**Affiliations:** ^1^ Plant Physiology, Bayreuth Center for Ecology and Environmental Research (BayCEER) University of Bayreuth Bayreuth Germany; ^2^ Environmental Geochemistry, Bayreuth Center for Ecology and Environmental Research (BayCEER) University of Bayreuth Bayreuth Germany

**Keywords:** arsenic speciation, arsenic toxicity, arsenic transport, thioarsenates, thiols

## Abstract

Arsenic (As) is one of the most problematic environmental toxins. Exposure to As, predominantly via drinking water and the intake of food, represents a major human health threat. Various species of As exist in the environment, among them organic and inorganic thioarsenates. Their ubiquitous presence in rice paddy soil pore water has recently been established. Thioarsenates are taken up by plants and show high mobility within plants. They are efficiently translocated from roots to shoots and can be loaded into grains. To date, however, no information is available on the transporter proteins enabling the necessary membrane passages. We tested the hypothesis that the major inorganic thioarsenate, monothioarsenate (MTA), is a substrate for phosphate transporters in experiments with yeast and plant model systems. Short‐term uptake assays demonstrated MTA transport, albeit at much lower rates than apparent for arsenate. Plant mutants with defects in phosphate transporters or regulators controlling phosphate deficiency responses were more tolerant to MTA as indicated by growth phenotypes and pigment concentrations. High external phosphate supply suppressed the MTA effects. Also, the mutants accumulated less As in roots and shoots upon MTA exposure. Inside plants, MTA was efficiently converted into arsenite and activated the phytochelatin pathway. Nonetheless, in light of the much lower relative uptake rate for MTA, we hypothesize that this As species exerts specific toxicity effects.

## Summary statement

Thioarsenates are nearly ubiquitously present in the environment. They can enter plant roots and show high mobility within plants. The major inorganic thioarsenate, monothioarsenate, is taken up into cells by phosphate transporters.

## 1 Introduction

Arsenic (As) is part of the natural environment. Its concentration in the earth's crust is about 2 mg/kg (Zhu et al. 2014). Weathering can release As from minerals into soil or groundwater, thereby making it available for the interaction with organisms. Other processes such as volcanic emissions and geothermal activities contribute to As presence in the biosphere as well. In addition, there is anthropogenic As pollution stemming from, for example, spraying of As‐containing pesticides, the manufacturing of electronic devices (Zhu et al. 2014) or medical uses such as the treatment of promyelocytic leukemia with As trioxide (Chen and Costa 2021). Predominantly via the uptake and accumulation by plants, As enters food webs (Tang and Zhao 2021). When As is taken up by crop plants, in particular rice, it can add to human As exposure. Because As is potentially highly toxic, the chronic dietary intake even of trace amounts of As can threaten human health (Zhao et al. 2024). Arsenic is a class I carcinogen and has been linked to skin, bladder and lung cancer. Furthermore, associations of As exposure with elevated risk of cardiovascular disease have been established (Jomova et al. 2025). Because of such adverse health effects and the potential of human exposure, As ranks first on the ATSDR (Agency for Toxic Substances and Disease Registry) priority list of hazardous substances (ATSDR (Agency for Toxic Substances and Disease Registry 2024). Arsenic is not only highly toxic for humans. Elevated As in paddy fields can have negative effects on yield, e.g. shown in field trials in Bangladesh (Huhmann et al. 2017). Thus, both human health and agricultural thresholds for soil As are relevant. A recent analysis of global soil pollution calculated that 1.1% of all agricultural soils exceed at least one of these for arsenic (Hou et al. 2025).

Several different chemical forms of As are present in the environment. Among the inorganic As species, arsenate is dominant in aerated soil while arsenite is the major form under the low oxygen conditions of flooded soil. Microbial activity in the soil can result in the formation of the organic As species monomethylarsenate (MMA) and dimethylarsenate (DMA) (Zhu et al. 2014). All of these As species can be taken up adventitiously into plants by transporters for essential or beneficial mineral nutrients. Arsenate chemically resembles phosphate and can therefore pass through phosphate transporters. This was demonstrated for various organisms. In *Saccharomyces cerevisiae* the high‐affinity phosphate transporter PHO84 mediates arsenate uptake (Persson et al. 1998; Shen et al. 2012). Homologous genes were identified in several plant species and found to encode phosphate uptake systems able to transport arsenate as well. For example, Pht1;1 and Pht1;4 have major roles in phosphate acquisition by *Arabidopsis thaliana* roots. Mutants defective in these two transporters not only showed reduced phosphate accumulation but were also more arsenate tolerant (Shin et al. 2004). This indirectly supports arsenate transport activity. Similarly, an arsenate‐tolerant *A. thaliana* was found to carry a mutation in *Pht1;1* (Catarecha et al. 2007). Several other members of the Pht1 family were later indirectly implicated in arsenate uptake, too, again based on higher arsenate tolerance of mutant lines for the respective genes (Nagarajan et al. 2011; Remy et al. 2012). In rice, homologous proteins such as OsPT1 have been shown to account for arsenate uptake (Kamiya et al. 2013). Arsenite in flooded soil is mostly present in a non‐dissociated form at normal pH (Zhao et al. 2010). Its uptake into plant cells is mediated by aquaglyceroporins (Bienert et al. 2008; Pommerrenig et al. 2020). Well documented is, for example, a prominent role of the silicon transporter Lsi1 in arsenite uptake by rice (Ma et al. 2008). Evidence supporting a contribution to the uptake of methylated As species was reported as well (Li et al. 2009).

In recent years, the number of relevant As species detected in flooded soils has grown. Thioarsenates, originally identified in sulfide‐rich habitats such as geothermal waters (Planer‐Friedrich et al. 2007), were found to be nearly ubiquitously present in the porewater of rice paddy fields (Wang et al. 2020; Dai et al. 2021). Both inorganic and methylated forms exist. The latter arise from thiolation of methylated oxyarsenates through sulfide produced by sulfate‐reducing bacteria (Dai et al. 2021). Of particular concern from a human health perspective is dimethylmonothioarsenate (DMMTA), because several studies demonstrated a toxicity for human and other mammalian cells that is even higher than that of arsenite (Naranmandura et al. 2011).

All thioarsenates investigated to date can enter plant cells and are mobile within the plant. For example, *A. thaliana* seedlings exposed to monothioarsenate (MTA) showed toxicity symptoms such as root growth reduction. The IC_50_ values (concentrations causing a 50% growth reduction of seedlings) for arsenate and MTA were in a similar range (Planer‐Friedrich et al. 2017). Rice plants exposed to inorganic and methylated thioarsenates showed uptake into roots, the xylem sap and the shoot, albeit at different rates (Kerl et al. 2019). MTA, dithioarsenate (DTA), DMMTA and dimethyldithioarsenate (DMDTA) were even detected in commercial rice grains, i.e. in material not deliberately exposed to As (Colina Blanco et al. 2021). It is therefore clear that thioarsenates are transported across biological membranes. To date, however, no information on pathways and responsible proteins is available (Zhao et al. 2022).

In this study, we aimed at identifying transporters involved in the membrane passage of MTA. Because its structural analog is arsenate, we hypothesized that phosphate transporters may be responsible for MTA uptake. Several lines of evidence demonstrated that this is indeed the case. We show MTA uptake by *S. cerevisiae* wild‐type cells and a reduction of this activity in the *pho84* mutant. *A. thaliana* mutants carrying defects in genes encoding either a major phosphate transporter or transcription factors essential for the upregulation of P uptake systems are more MTA tolerant and accumulate less As upon MTA exposure. Arsenic speciation analysis indicated efficient reduction of MTA to arsenite. However, given the much lower rate of uptake a fraction of MTA appears to exert specific toxicity inside cells.

## 2 Materials and Methods

### 2.1 Arsenic Species

Arsenite was purchased from Riedel‐de Haёn, arsenate from Fluka Chemicals. MTA was synthesized by adding As_2_O_3_ and elemental sulfur to NaOH with a molar ratio of As: NaOH: S = 1: 3: 0.9, heating the solution to 100°C, and maintaining the temperature for 2 h. The MTA was crystallized from the solution by cooling at 4°C, and then dried and stored at 4°C. The purity of the synthesized MTA (molar ratio of MTA/total As after dissolving the synthesized MTA in water) was 97.3% of total As with 0.7% arsenite and 2% arsenate.

### 2.2 Uptake Assays With Yeast

For uptake assays with *S. cerevisiae* WT (BY4742) and the *pho84* mutant strain, cells were cultivated in complete YPD medium at 30°C and 220 rpm. Log‐phase cultures were collected by centrifugation, washed twice with sterile water, and resuspended in an uptake solution (10 mM Hepes/Tris, 0.1 mM MgCl_2_, 2% w/v glucose). A 40 ml aliquot was taken from the resuspended cells before dividing them into three treatment groups. Uptake experiments were initiated by adding 50 µM MTA, arsenate, or arsenite. Samples were taken at indicated time points. Cells were harvested by centrifugation and washed again. The pellets were dried at 60°C for 32 h prior to microwave‐digestion and determination of total As accumulation.

### 2.3 Plant Genotypes and Cultivation

Experiments were performed with *A. thaliana* wild‐type Col‐0 and the mutants *pht1;1* (SALK_088586) and *phr1phl1‐1* (Bustos et al. 2010). Plants were grown hydroponically, on vertical agar plates or in a liquid seedling assay (Pischke et al. 2022) in modified 1/10 Hoagland medium (0.25 mM KNO_3_, 0.1 mM MgSO_4_, 0.1 mM Ca(NO_3_)_2_, 0.25 mM KH_2_PO_4_, 1.75 µM Fe‐HEBED, 3.5 µM H_3_BO_3_, 0.7 µM MnCl_2_, 25 nM CuSO_4_, 50 nM ZnSO_4_, 10 nM NaMoO_4_, 0.5 µM NaCl, 0.5 nM CoCl_2_, 2 mM MES (pH 6.0). KH_2_PO_4_ concentrations were modified as indicated. For root growth and biomass analysis, seeds were sown on plates containing 55 µM KH_2_PO_4_ and 0.8% (w/v) plant agar (Duchefa). Seedlings were grown under long‐day conditions for 14 to 18 days in the presence or absence of MTA or arsenate. Root lengths and fresh weights were recorded for each seedling. In liquid seedling assays, seeds were placed into individual wells of a six‐well plate and cultivated under long‐day conditions (16 h light/8 h dark, 23°C, ~100 µE) with gentle shaking for 7 days after which fresh weight measurements were taken for all seedlings in each well and normalized to the total number of seedlings. For hydroponic culture sterilized seeds were placed on PCR tubes in tip boxes containing medium supplemented with 250 µM KH_2_PO_4_. After a 2‐day stratification, the boxes were transferred and seedlings were cultivated for 3 ‐ 4 weeks under short‐day conditions at 23°C (8 h light/16 h dark, 23°C, ~100 µE) before being transferred to 50 ml Falcon tubes, where they were cultivated under the same conditions. Prior to treatments, plants were cultivated in medium containing 2 µM phosphate for 3 days, followed by phosphate‐free medium for 1 day. For the treatment, seedlings were placed in P_i_‐free medium with or without MTA or arsenate. After 24 h, plants were harvested, separated into roots and shoots, and washed for 10 min in 1 mM KH_2_PO_4_, 5 mM Ca(NO_3_)_2_, and 5 mM MES (Xu et al. 2007). Depending on the analysis, plant material was either dried at 60°C for 7 days to determine As content or flash‐frozen in liquid nitrogen for arsenic speciation analysis.

### 2.4 Chlorophyll and Cyanidin Analysis

Depending on the treatment, 5–10 shoots per genotype were collected and their fresh weight was determined. The harvested plant material was immediately flash‐frozen in liquid nitrogen and stored at −80°C for further analysis. Chlorophyll content was quantified following the method of Ticconi et al. (Ticconi et al. 2001), based on Chapman et al. (Lobban et al. 1988), while cyanidin levels were measured as described by Li et al. (Li et al. 2023).

### 2.5 Thiol Analysis

Harvested seedlings were separated into roots and shoots, weighed for fresh biomass determination, flash‐frozen in liquid nitrogen and stored at −80°C. To extract thiol compounds, three volumes of extraction buffer (0.1% (v/v) trifluoroacetic acid, 6.3 mM diethylenetriamine pentaacetic acid (DTPA), and 0.04 mM N‐acetylcysteine (internal standard) were added per mg of fresh plant material. For derivatization, a mix of 200 mM EPPS containing 6.3 mM DTPA (pH 8.2, adjusted with NaOH) and 3.13 µL of tris(2‐carboxyethyl)phosphine (dissolved in 200 mM EPPS, pH 8.2) was added. The labeling reaction was initiated by adding 50 mM monobromobimane (dissolved in acetonitrile) and stopped after 30 min incubation at 45°C, by adding 12.5 µl of 1 M methanesulfonic acid. Samples were stored at −20°C until HPLC analysis. Thiol profiles were recorded as described (Fischer et al. 2014).

### 2.6 Determination of Total as and Analysis of as Speciation

The arsenic content in digested *S. cerevisiae* and *A. thaliana* samples was quantified using an inductively coupled plasma mass spectrometer (ICP‐MS), 8900 ICP – Triple Quad (Agilent), with oxygen as reaction cell gas (AsO^+^, m/z 75 → 91). Arsenic species were detected as AsO⁺ at m/z 91, with rhodium (Rh⁺, m/z 103) serving as the internal standard. A certified reference material (TMDA 54.6, Canada) was used for quality control. Arsenic species in plant tissues and medium samples were separated by ion chromatography (IC, 940 Professional IC Vario Metrohm) and quantified through ICP‐MS. For the chromatographic separation, an AS16 column (Dionex AG/AS16 IonPac) was used, with a 2.5–100 mM NaOH gradient, 1.2 mL min^–1^ flow rate, and an injection volume of 50 μL (Wallschläger and London 2008).

### 2.7 Statistical Analysis

Statistical significance between treatments was assessed using the Kruskal‐Wallis test, followed by a post hoc test for pairwise comparisons (*p* < 0.05). In the case of *S. cerevisiae* uptake experiments and the analysis of chlorophyll and anthocyanin contents in *A. thaliana*, a two‐sample *t*‐test with pooled variance was used to compare genotypes within each treatment. Data are depicted as the mean ± standard deviation.

## 3 Results

**Figure 1:**
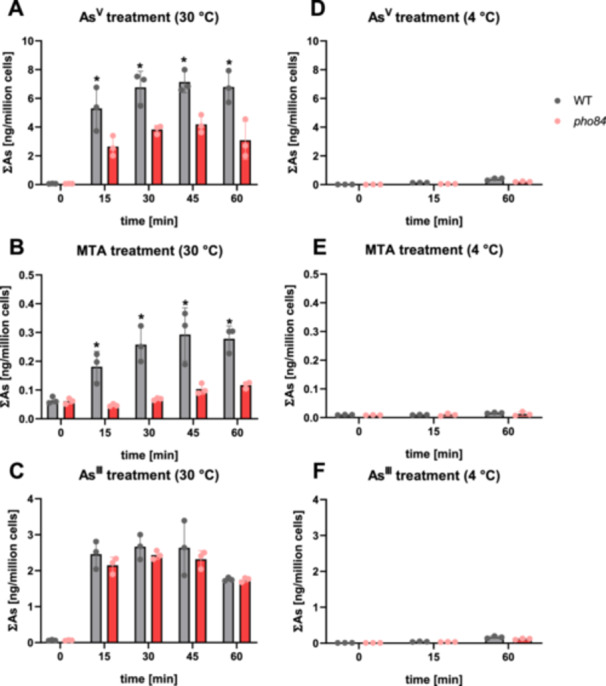
Short‐term uptake of different As species by *S. cerevisiae* BY4742 WT and the *pho84* mutant under P_i_‐deplete conditions. Logarithmically growing cells were transferred to an uptake solution containing 50 µM arsenate (As^V^), arsenite (As^III^) or MTA. Cells were harvested after different time points of incubation at 30°C (A–C), or at 4°C (D–F). Total As (∑As) in *S. cerevisiae* cells was quantified using an ICP‐MS and normalized to the OD_600_ value measured at each time point. Statistical analysis was performed using the two‐sample *t*‐test for pooled variance among the genotypes for each treatment and time point separately. Statistically significant differences (*p* < 0.05) are indicated by an asterisk (*). Data represent the mean ± standard deviation of three independent biological replicates (*n* = 3).

As an initial test of the hypothesis that MTA is taken up into cells by phosphate transporters, short‐term uptake assays were performed with *S. cerevisiae* wild‐type and *pho84* cells. First, arsenate uptake was monitored. Results confirmed the known contribution of PHO84 to arsenate uptake as mutant cells showed only about half the As accumulation of wild‐type cells at all time points (Figure 1A). Accumulation at 4°C was negligible for all tested As species, confirming protein‐dependent uptake (Figure 1D–F). In contrast to arsenate, there was no genotype effect on As accumulation in cells treated with arsenite, demonstrating the specificity of PHO84‐dependent As transport. When cells were exposed to MTA, an increase in As accumulation over 45 min was observed for wild‐type cells. Overall, the uptake rate was about 20‐fold lower than for arsenate. Importantly, the *pho84* mutant showed strongly reduced MTA uptake (Figure 1C). Speciation analysis of the yeast uptake solution confirmed that conversion of arsenate or MTA to arsenite was negligible (arsenate: 0.162%/0.096%; MTA: 0.049%/0.079%) (Supplementary Figure S1).

**Figure 2:**
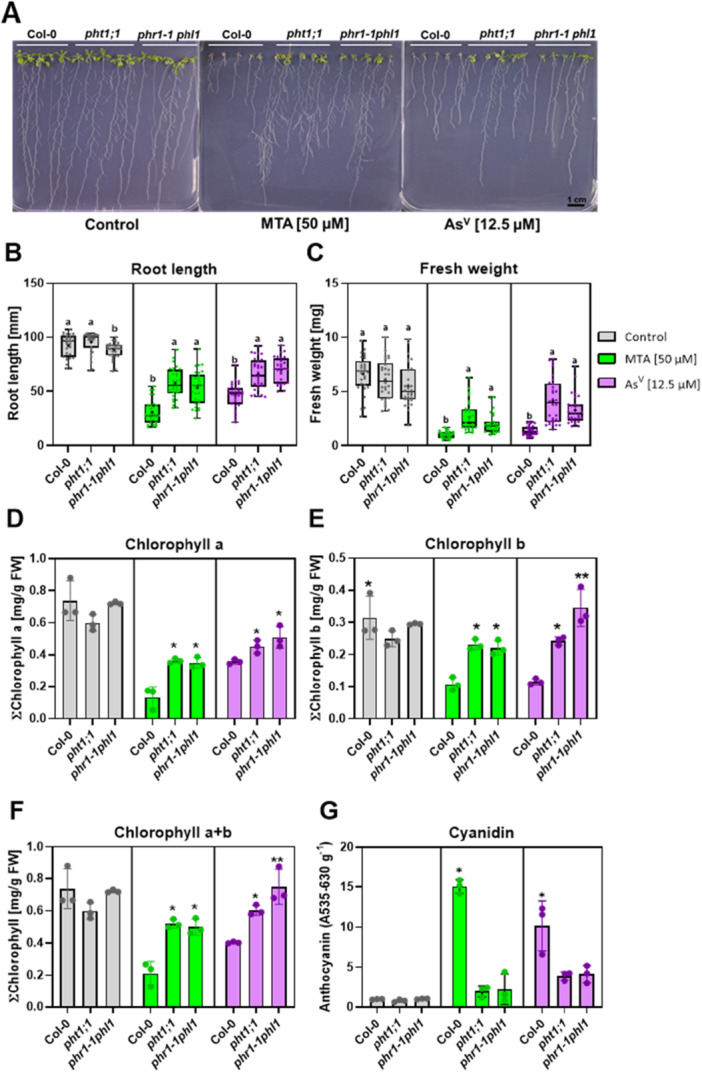
Loss of PHT1;1 and an impaired P_i_ starvation response enhance tolerance to MTA. Seedlings of WT (Col‐0), the *pht1;1* mutant and the *phr1‐1phl1* mutant were grown on plates either under control conditions, with 50 µM MTA or 12.5 µM arsenate (As^V^) (A). Root lengths (B) and fresh weights (C) were determined after 14 days (three independent biological replicates; *n* = 28–30 per condition and genotype; mean values are marked with a cross symbol). Statistical analysis of growth was performed using the Kruskal‐Wallis test to assess significant differences among genotypes for each treatment separately, followed by a post hoc test for pairwise comparisons. Significant differences (*p* < 0.05) are indicated by different letters. Also, chlorophyll a (D), chlorophyll b (E), and ∑chlorophyll concentrations (F) were measured for each treatment, shown as bar plots with individual data points for each replicate. Anthocyanin accumulation was quantified through cyanidin levels (G). Data represent three independent biological replicates. Statistical analysis of pigment data was performed using a two‐sample *t*‐test with pooled variance to assess significant differences in chlorophyll and anthocyanin contents between genotypes through pairwise comparisons for each treatment. Significant differences (*p* < 0.05) are marked with an asterisk (*). Data represent the mean ± standard deviation of three biological replicates (*n* = 3) (D–G) or max to min (B, C). [Color figure can be viewed at wileyonlinelibrary.com]

Having established MTA transport by phosphate transporters, we turned to analyses using the model plant *A. thaliana*, which offers ease of experimentation and, more importantly, a wealth of suitable and accessible mutants. Previous investigations of plant arsenate uptake had mostly relied on tolerance assays with mutants affected in phosphate uptake. Accordingly, we chose the *pht1;1* mutant lacking a major root uptake system (Shin et al. 2004), and the regulatory double mutant *phr1phl1* which is unable to mount an efficient upregulation of phosphate uptake capacity under conditions of phosphate deficiency (Bustos et al. 2010). Seedlings were cultivated in low phosphate conditions and exposed to either arsenate or MTA. Concentrations were chosen so that the effects of arsenate and MTA on wild‐type seedlings were comparable. While no differences between the genotypes were observed in root length or seedling fresh weight under control conditions, the two mutant lines grew significantly better than wild type in the presence of either arsenate or MTA (Figure 2A–C). Also, chlorophyll concentrations were higher in mutant seedlings than Col‐0 after treatment (Figure 2D–F). Conversely, the accumulation of anthocyanins, which is a stress indicator (Yan et al. 2022), was strongly elevated in wild‐type seedlings compared to mutant seedlings (Figure 2G). Increasing P_i_ concentrations in the medium suppressed the differences in arsenate and MTA tolerance between Col‐0 and the mutant lines (Supplementary Figure S2), consistent with competition between arsenate or MTA and phosphate for uptake. The higher tolerance of the phosphate transport and the phosphate starvation response mutant were in addition tested in a liquid seedling assay. Again, the wild type was more strongly affected by both arsenate and MTA while no differences in growth were detected under control conditions (Supplementary Figure S3).

**Figure 3:**
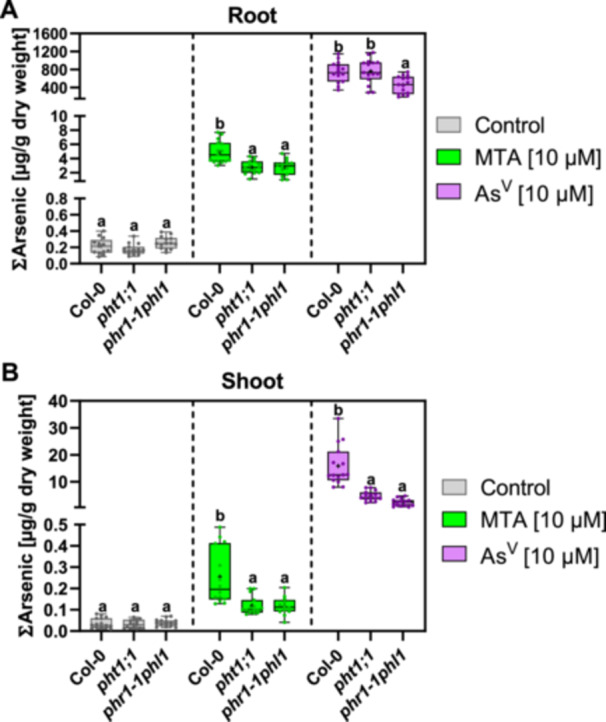
Mutants with reduced phosphate uptake capacity accumulate less As in roots and shoots after exposure to MTA. Five‐ to six‐week‐old hydroponically grown Col‐0, *pht1;1*, and *phr1‐1phl1* plants were transferred to medium containing 2 µM P_i_ for three days and then to P_i_‐free medium one day prior to the exposure, to induce phosphate starvation responses. Plants were treated with MTA or arsenate (As^V^) for 24 h in P_i_‐free medium. The total as (∑As) of roots (A) and shoots (B) was quantified by ICP‐MS. Statistical analysis was performed using the Kruskal‐Wallis test to assess significant differences in ∑As between genotypes for each treatment separately, followed by a post hoc test for pairwise comparisons. Statistically significant differences (*p* < 0.05) are indicated by different letters. Mean values are marked with a cross symbol. Data represent three independent biological experiments; *n* = 13–15 per condition and genotype. [Color figure can be viewed at wileyonlinelibrary.com]

The tolerance phenotypes had indicated that phosphate uptake pathways mediate the entry not only of arsenate into plants but of MTA as well. We therefore tested As accumulation directly and grew plants hydroponically for five to 6 weeks to obtain enough root biomass for reliable elemental and speciation analysis. Wild‐type roots took up arsenate much more efficiently than MTA. Total As (∑As) after 24 h exposure was more than 100fold higher in arsenate‐treated plants than in MTA‐treated plants even though equal concentrations (10 µM) were applied (Figure 3A). Translocation of As to the shoot was about 60fold more efficient after arsenate than after MTA exposure (Figure 3B). Importantly, the *pht1;1* and the *phr1phl1* mutant accumulated significantly less As in roots than wild type when exposed to MTA. For arsenate‐treated plants, the difference was significant only for *phr1phl1* (Figure 3A). Translocation of As to the shoot was significantly lower than wild type in both mutant lines (Figure 3B).

**Figure 4:**
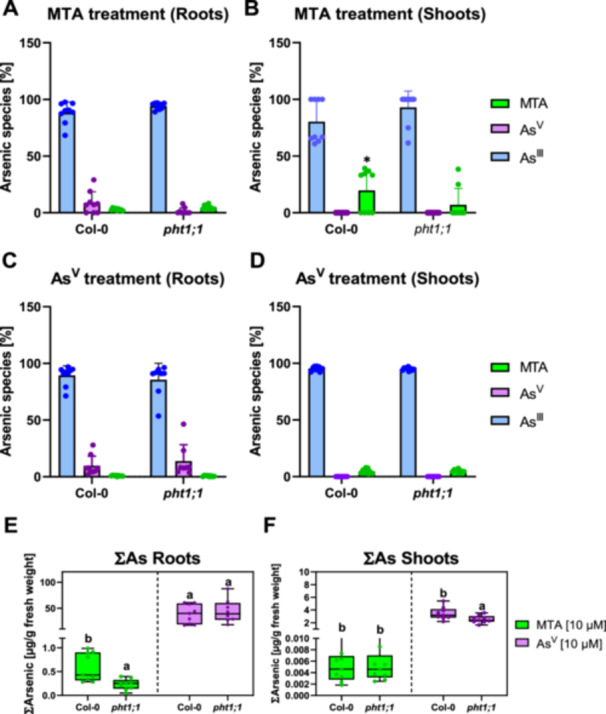
MTA is efficiently reduced to arsenite in WT (Col‐0) and *pht1;1* mutant plants. Five‐ to six‐week‐old hydroponically grown Col‐0 and *pht1;1* plants were transferred to medium containing 2 µM P_i_ for three days and then to P_i_‐free medium one day prior to the exposure, to induce phosphate starvation responses. Plants were treated with MTA or arsenate (As^V^) as indicated for 24 h in P_i_‐free medium. Arsenic speciation was performed by IC‐ICP‐MS. The percentage share of the different As species in roots and shoots of each genotype is shown in A–D, the respective total As (∑As) in E and F. Data represent nine root (A + C) and shoot samples (B + D) per treatment and genotype (three independent experiments). Shown are means ± standard deviation (A–D) or max to min (E and F, mean values are marked with a cross symbol). Statistical analysis was performed using the Kruskal‐Wallis test to assess significant differences in ∑As among genotypes for each treatment separately, followed by a post hoc test for pairwise comparisons. Significant differences (*p* < 0.05) are indicated by letters (∑As) or with an asterisk (*) (E + F). [Color figure can be viewed at wileyonlinelibrary.com]

Arsenic speciation analysis was exemplarily performed for roots and shoots of Col‐0 and *pht1;1*. It showed that the majority of MTA and arsenate was efficiently reduced to arsenite after uptake into cells. The latter accounted for over 80% of total As (∑As) after MTA or arsenate treatment (Figure 4A,C). Data for shoots were very similar with the exceptions that conversion of MTA to arsenite was slightly less efficient and Col‐0 contained more MTA than the *pht1;1* mutant (Figure 4B,D). Interestingly, the shoots of arsenate‐exposed plants contained small quantities of MTA, accounting for approximately 2.4% to 7.9% of ∑As.

**Figure 5:**
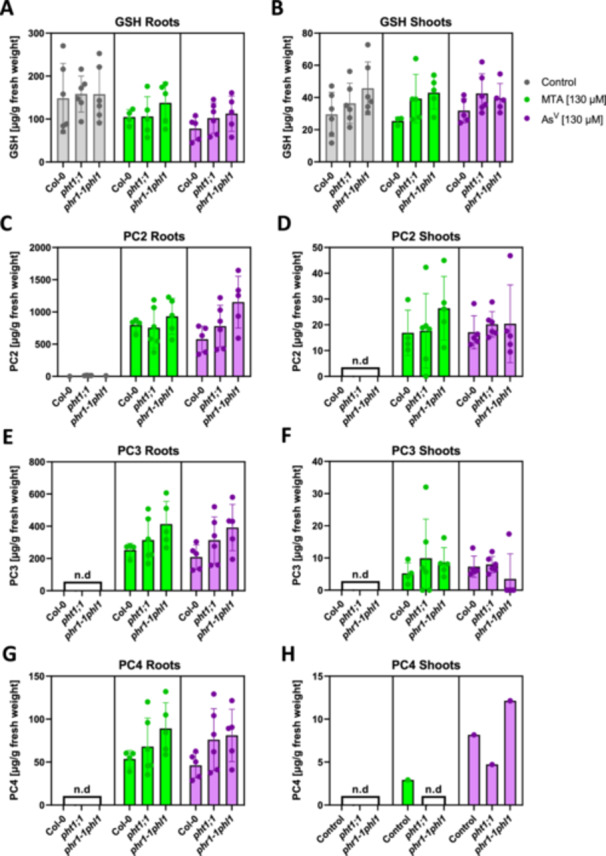
Phytochelatin accumulation in arsenate‐ and MTA‐treated seedlings. Seedlings of WT (Col‐0), the *pht1;1* mutant and the *phr1‐1phl1* mutant were grown under control conditions or on plates with either arsenate or MTA (130 µM). Thiol profiles (GSH, A, B; PC2, C, D; PC3, E, F; PC4, G, H) of roots (left) and shoots (right) of Col‐0, *pht1;1* and *phr1‐1phl1* seedlings were assessed after 72 h of exposure to MTA or arsenate (As^V^). Data represent the mean ± standard deviation of three independent biological replicates; 34–36 individual seedlings per condition and genotype were pooled; n.d. = not detectable. Statistical analysis was performed using the Kruskal Wallis test. No significant differences between genotypes were detected. [Color figure can be viewed at wileyonlinelibrary.com]

The detoxification of inorganic As in plants depends on the phytochelatin pathway (Clemens and Ma 2016). Arsenite activates phytochelatin synthases (PCS) in the cytosol. PCS then synthesize phytochelatins from glutathione. Monitoring PC accumulation can therefore be used to indirectly analyze cytosolic As speciation. When plants were treated with arsenate or MTA – both at higher concentrations to facilitate detection of longer‐chain PCs – pronounced PC formation was detected in roots and shoots (Figure 5), confirming the speciation results, namely the efficient reduction of arsenate and MTA to arsenite (Figure 4). Accumulation of PC2, PC3 and PC4 was much stronger in roots than in shoots, which is consistent with the difference in total As content between roots and shoots (Figure 3). No significant differences were observed between arsenate and MTA treatment or between the three genotypes.

## 4 Discussion

Inorganic and methylated thioarsenates have in recent years been found to be ubiquitously present in rice paddy soils. MTA is the most stable thiolated species, occurring over a wide pH range, and is therefore prominent among inorganic thioarsenates (Wang et al. 2020). Under sulfate‐reducing conditions it arises spontaneously from arsenite by OH − /SH− ligand exchange and oxidative addition of elemental sulfur (Stauder et al. 2005). Studies with rice and *A. thaliana* showed that MTA is taken up by plants, exerts toxic effects and is mobile within the plant (Planer‐Friedrich et al. 2017; Kerl et al. 2018), reaching the grain even of rice plants grown under regular field conditions, i.e. in the absence of any contamination or deliberate exposure (Colina Blanco et al. 2021). Transporters mediating the passage of MTA or other thioarsenates through biological membranes, however, are not known to date. Identifying them may provide opportunities to lower As accumulation through engineering approaches (Clemens 2019; Zhao et al. 2022).

Toxic metalloids and metals without a biological function are taken up adventitiously, because nutrient transporter proteins do not discriminate perfectly between chemically similar substrates. MTA resembles arsenate with respect to its tetrahedral structure and the relevant *p*K_a_, *p*K_2_ (Planer‐Friedrich et al. 2017). Plant arsenate uptake via phosphate transporters is well established (Meharg and Macnair 1992; Shin et al. 2004; Castrillo et al. 2013; Wang et al. 2016). Based on this evidence we hypothesized that MTA would be transported by phosphate transporters as well.

In unicellular model organisms the connection between phosphate transporters and arsenate uptake had been shown earlier (Garbinski et al. 2019). *E. coli* expresses a low‐affinity phosphate uptake system, Pit, which allows entry of arsenate, too (Rosenberg et al. 1977). *S. cerevisiae* PHO84 plays a similar role (Bun‐ya et al. 1996). We used a *pho84* mutant to test MTA transport directly. Short‐term uptake assays demonstrated higher arsenate uptake in the wild type than the mutant, confirming earlier work. For arsenite no difference between the two strains was detected, which evidenced specificity of As transport. MTA‐treated cells showed an even stronger genotype‐dependent difference than arsenate‐treated cells. The strain lacking functional PHO84 contained around 75% less As at the earlier time points and ca. 50% less at the end of the experiment relative to the wild type (Figure 1). Thus, PHO84 allows entry of MTA, albeit at much lower rates than apparent for arsenate.

Having established that MTA can enter cells through phosphate transporters, we selected well‐characterized *A. thaliana* mutants and the respective wild type Col‐0 to obtain insights into MTA uptake by plants. Pht1;1 is one of several root‐expressed transporters in the Pht1 family homologous to PHO84. Transcript abundance increases strongly in phosphate‐limited conditions. Mutant analysis revealed a major contribution of Pht1;1 to phosphate uptake by *A. thaliana* roots under both phosphate‐deplete and ‐replete conditions (Shin et al. 2004). A recent GWAS analysis of phosphate acquisition further supported the dominant role of Pht1;1. *Pht1;1* haplotypes were found to be associated with either high or low phosphate uptake activity and occurrence of *A. thaliana* accessions in either low phosphate or high phosphate soil, respectively (Chien et al. 2022). The transcription factors PHR1 and PHL1 represent master regulators of the extensive phosphate starvation response in *A. thaliana*. Loss of the two partially redundant genes strongly impairs the ability to mount a transcriptional reprogramming under low phosphate conditions and affects both phosphate uptake and root development (Bustos et al. 2010).

Arsenate exposure experiments with these mutants confirmed the role of phosphate transporters in arsenate uptake. First, the two mutant lines showed higher arsenate tolerance apparent from greater root length and higher seedling fresh weights compared to Col‐0 (Figure 2, Supplementary Figure S3). These phenotypes could be suppressed by elevated phosphate concentrations in the medium (Supplementary Figure S2). Also, the mutant leaves were less chlorotic, quantified as chlorophyll concentrations, and showed strongly reduced accumulation of anthocyanins (Figure 2). With respect to the latter there was no difference between the two mutants, suggesting that the much lower anthocyanin content was not due to the known defect of the *phr1phl1* mutant in anthocyanin synthesis under phosphate starvation conditions (Bustos et al. 2010). Second, As accumulation in the shoot was significantly reduced in both mutants, and root As content was lower in *phr1phl1* (Figure 3).

When Col‐0 and the mutant lines were treated with an MTA concentration causing effects equal to those of 10 µM arsenate, the two mutant lines were again less affected than the wild type in low phosphate medium but not in the presence of high phosphate. Root lengths and seedling fresh weights as well as chlorophyll concentrations were higher. Conversely, anthocyanin accumulation was lower (Figure 2; Supplementary Figure. S2). These observations are consistent with MTA uptake via phosphate transporters. Correspondingly, total As was significantly lower in roots and shoots of both mutants. This effect was more pronounced than in arsenate‐treated plants. Furthermore, there was no difference between the mutant lines, suggesting that Pht1;1 accounts for essentially all Pht1‐dependent MTA uptake while in the case of arsenate, other Pht1 family members contribute too (Shin et al. 2004). This may be attributable to a divergence in relative affinities for MTA and arsenate.

Similar to yeast, plants took up arsenate much more efficiently than MTA. In our experiments, Col‐0 roots accumulated around 800 µg As g^‐1^ d.w. after 24 h of exposure to 10 µM arsenate. This value is in the range of previously reported data (Sánchez‐Bermejo et al. 2014) and about 150fold higher than determined for MTA‐treated roots at the same external concentration. Previous studies with rice plants had already indicated lower efficiency of MTA uptake relative to arsenate, albeit with a less extreme difference (Kerl et al. 2018). Respective experiments with wild‐type rice and suitable phosphate transport mutants will be needed to corroborate the findings on MTA uptake, toxicity and in planta conversion reported here for *A. thaliana*. Regardless of the actual MTA uptake rate, however, detection of MTA in commercial rice grains (Colina Blanco et al 2021) strongly suggests that MTA does indeed enter rice plants under field conditions.

Transport to the shoot was slightly more efficient following MTA treatment. Translocation factors were ca. 0.025 for arsenate and ca. 0.06 for MTA. This minor variance suggested similar fates of arsenate and MTA inside the plant. Indeed, speciation analysis indicated nearly quantitative conversion of both arsenate and MTA to arsenite (Figure 4). Efficient reduction to arsenite after uptake is well‐documented for arsenate (Sánchez‐Bermejo et al. 2014; Chao et al. 2014) and, according to our data, the same applies to MTA. The initial study on MTA exposure of *A. thaliana* had already suggested that MTA is reduced to arsenite (Planer‐Friedrich et al. 2017). Here this was shown directly for root and shoot tissue. Phytochelatin accumulation in arsenate‐ and MTA‐treated seedlings confirmed the speciation analysis. Activation of PC synthesis requires the formation of arsenite, thus the substantial synthesis of PC2, PC3 and PC4 indicates arsenite formation.

Considering the differences in uptake rate and As accumulation following arsenate versus MTA exposure, together with the efficient reduction to arsenite and similar levels of PC formation, two questions arise. First, why is there practically no difference in PC accumulation even though total As is much lower in MTA‐exposed plants? Second, why do arsenate and MTA show similar apparent toxicity when both are converted to arsenite, yet taken up at very different rates? Concerning the former, a possible explanation is saturation of PC synthesis capacity under our experimental conditions at least in roots. With respect to the latter, arsenate is efficiently reduced to arsenite in the outer cell layers of the root. This was shown by expressing the major arsenate reductase, HAC1, under control of cell type‐specific promoters in *hac1* mutant background (Fischer et al. 2021). Conversely, there are parts of the root, the stele in particular, where HAC1 function is impaired, presumably because other as yet unidentified components or conditions required for reduction are absent. It is currently unknown, whether these findings would apply to MTA in the same way. From the available speciation data, we cannot infer the kinetics or the specific localization of MTA conversion to arsenite. Possibly, MTA reduction proceeds less efficiently. MTA itself may then exert toxic effects in root cells before it is converted to arsenite. The fraction of MTA detectable in shoots after MTA exposure (Figure 4B) is much higher than the residual arsenate following arsenate exposure (Figure 4D). Such difference is consistent with the hypothesis that MTA is more likely than arsenate to escape reduction to arsenite. Also, it may indicate specific, and possibly much stronger, toxicity of MTA. Alternatively, the sulfur release that is assumed to occur in the course of MTA reduction to arsenite (Fisher et al. 2008) may trigger detrimental processes. Addressing these questions will require As speciation and metabolite analyses with ideally cellular resolution.

## Conflicts of Interest

Stephan Clemens is an Editorial Board member of Plant, Cell, and Environment and a coauthor of this article. To minimize bias, he was excluded from all editorial decision‐making related to acceptance of this article for publication.

## Supporting information


**Figure S1:** Stability of arsenic species during *S. cerevisiae* uptake experiments. **Figure S2:** High external phosphate concentrations suppress MTA toxicity. **Figure S3:** Loss of *PHT1;1* and an impaired Pi starvation response enhance tolerance to MTA in a liquid seedling assay.

## Data Availability

The data that support the findings of this study are available from the corresponding author upon reasonable request.
