# Supplementary material for: Phosphate Transporters Mediate the Uptake of Monothioarsenate
Source: Plant Cell Environ. 2026 Jan 28;49(5):2628–38. doi: 10.1111/pce.70400 (PMC13051750; doi:10.1111/pce.70400)
Supplement: Supplementary file 1 — Figure S1: Stability of arsenic species during S. cerevisiae uptake experiments. Figure S2: High external phosphate concentrations suppress MTA toxicity. Figure S3: Loss of PHT1;1 and an impaired Pi starvation response enhance tolerance to MTA in a liquid seedling assay. [file PCE-49-2628-s001.pdf]

Supplementary Material

Phosphate transporters mediate the uptake of monothioarsenate

Sebastian Haider[1], Sylvia Hafner[2], Britta Planer-Friedrich[2], Stephan Clemens[1]

[1] Plant Physiology, Bayreuth Center for Ecology and Environmental Research (BayCEER), University of Bayreuth, D-95440 Bayreuth, Germany

[2] Environmental Geochemistry, Bayreuth Center for Ecology and Environmental Research (BayCEER), University of Bayreuth, D-95440 Bayreuth, Germany

* Corresponding author phone: +49 921 55 2630, Email: stephan.clemens@uni-bayreuth.de

(4 pages, 3 figures)

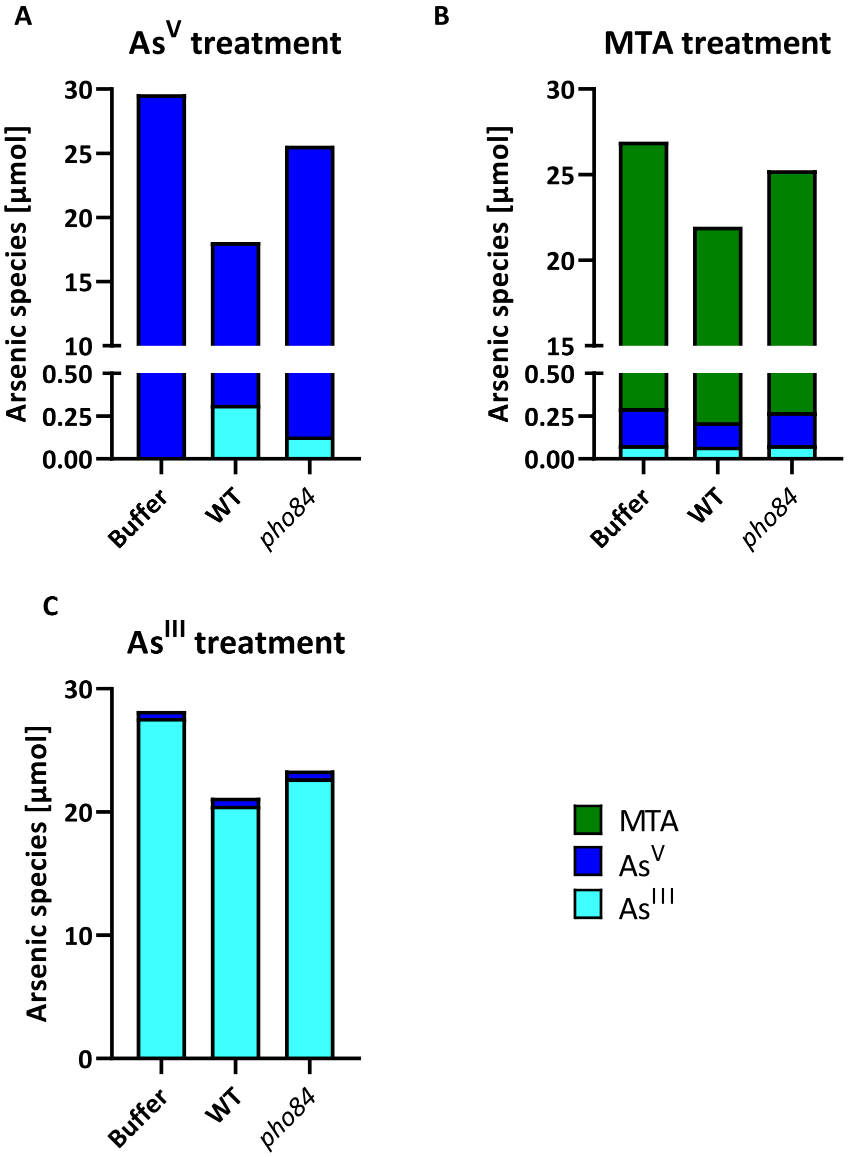

Fig. S1: Stability of arsenic species during *S. cerevisiae* uptake experiments. Samples were taken from uptake solutions of assays with *S. cerevisiae* WT and *pho84* cells after exposure to 50 µM arsenate (As^{V}), MTA, or arsenite (As^{III}) for 60 min at 30°C (one of the three replicates of the uptake experiments shown in Fig. 4.). Arsenic speciation was analyzed by IC-ICP-MS.

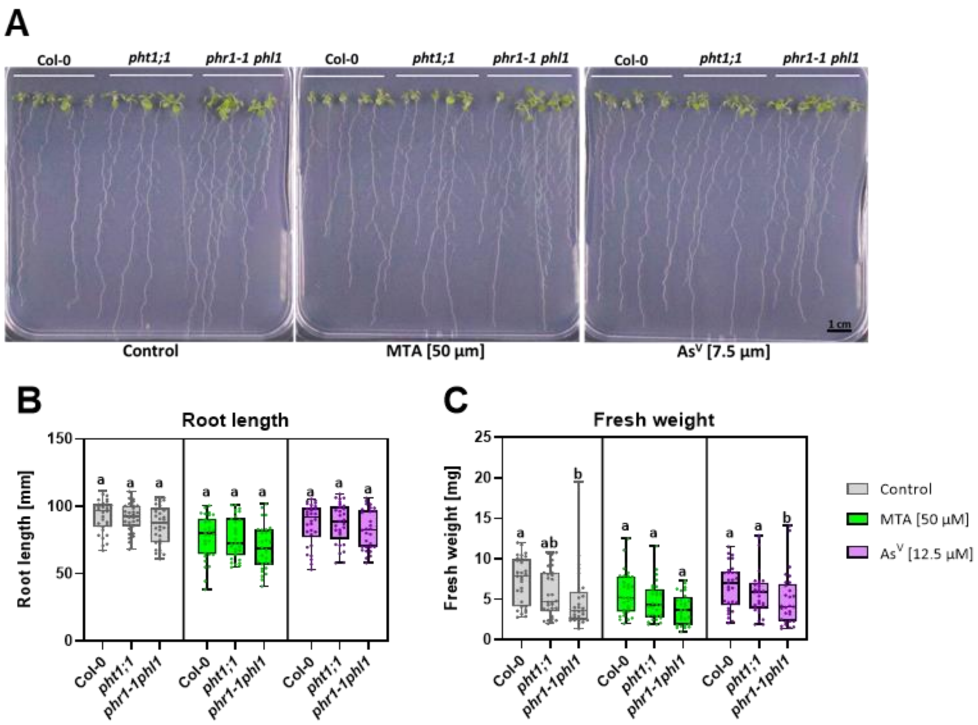

Fig. S2: High external phosphate concentrations suppress MTA toxicity. Seedlings of WT (Col-0), the *pht1;1* mutant and the *phr1-1phl1* mutant were grown either under control conditions, on plates with 50 µM MTA or 12.5 µM arsenate (As^V) in phosphate-replete medium (2.5 mM P_i)(A). Root lengths (B) and fresh weights (C) were determined after 14 days (three independent biological replicates; n = 28-30 per condition and genotype; medians are marked with a horizontal line). Statistical analysis of growth was performed using the Kruskal-Wallis test to assess significant differences among genotypes for each treatment separately, followed by a post hoc test for pairwise comparisons. Significant differences ($p < 0.05$) are indicated by different letters.

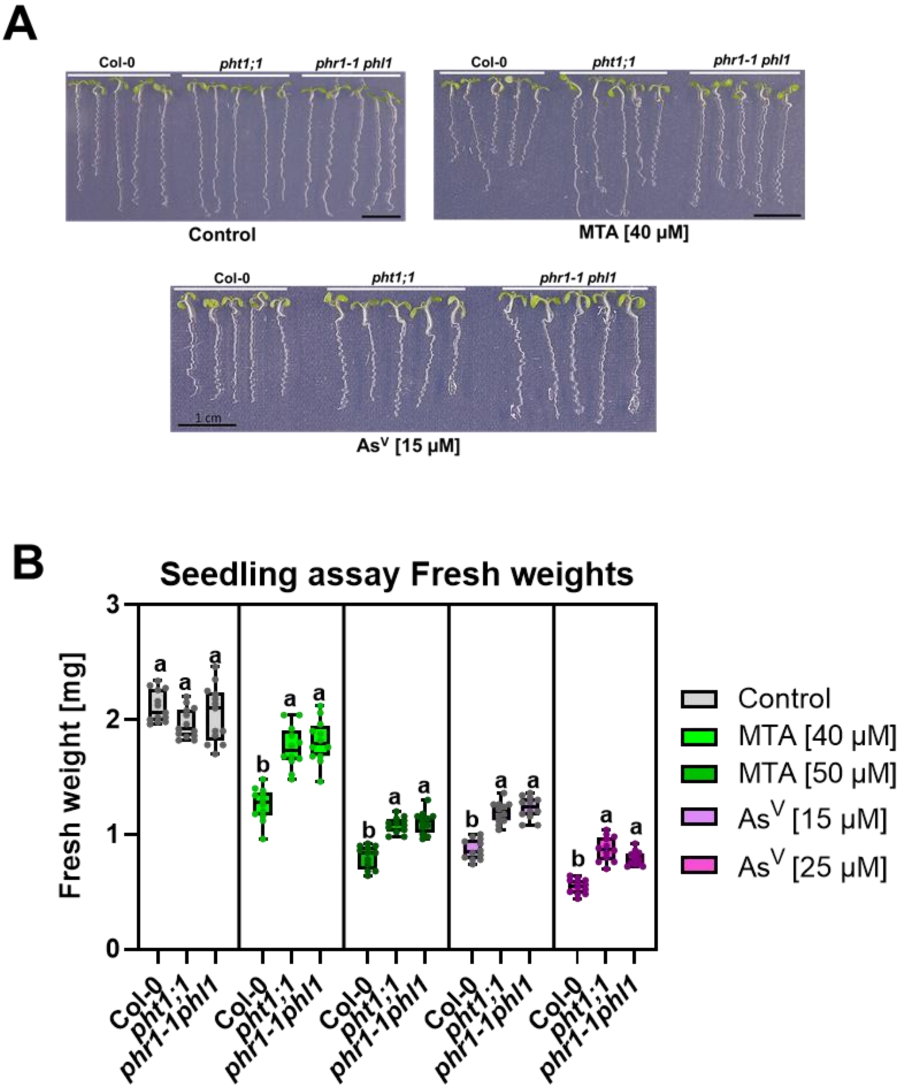

Fig. S3: Loss of PHT1;1 and an impaired P_i starvation response enhance tolerance to MTA in a liquid seedling assay. Seedlings of WT (Col-0), the *pht1;1* mutant and the *phr1-1phl1* mutant were grown in liquid medium under control conditions, or in the presence of two different MTA or arsenate (As^V) concentrations (40 and 50 µM, 15 and 25 µM, respectively). Root lengths (A) and fresh weights (B) were determined after 7 days (three independent biological replicates; n = 18-20 per condition and genotype, medians are marked with a horizontal line). Statistical analysis of growth was performed using the Kruskal-Wallis test to assess significant differences among genotypes for each treatment separately, followed by a post hoc test for pairwise comparisons. Significant differences ($p < 0.05$) are indicated by different letters.
